# Supplementary material for: Structural basis for the simultaneous recognition of NEMO and acceptor ubiquitin by the HOIP NZF1 domain
Source: Sci Rep. 2022 Jul 18;12:12241. doi: 10.1038/s41598-022-16193-4 (PMC9294000; doi:10.1038/s41598-022-16193-4)
Supplement: Supplementary file 1 — Supplementary Figures. [file 41598_2022_16193_MOESM1_ESM.pdf]

Structural basis for the simultaneous recognition of NEMO and acceptor ubiquitin by the HOIP NZF1 domain

Simin Rahighi[1]*, Mamta Iyer[1], Hamid Oveisi[1,2], Sammy Nasser[1], Vincent Duong[1]

[1]Chapman University School of Pharmacy (CUSP), Harry and Diane Rinker Health Science Campus, Chapman University, Irvine, CA 92618, USA

[2] Department of Materials and Polymer Engineering, Hakim Sabzevari University, Sabzevar 9617976478, Iran

* Correspondence should be addressed to: rahighi@chapman.edu

Supplementary Figure S1

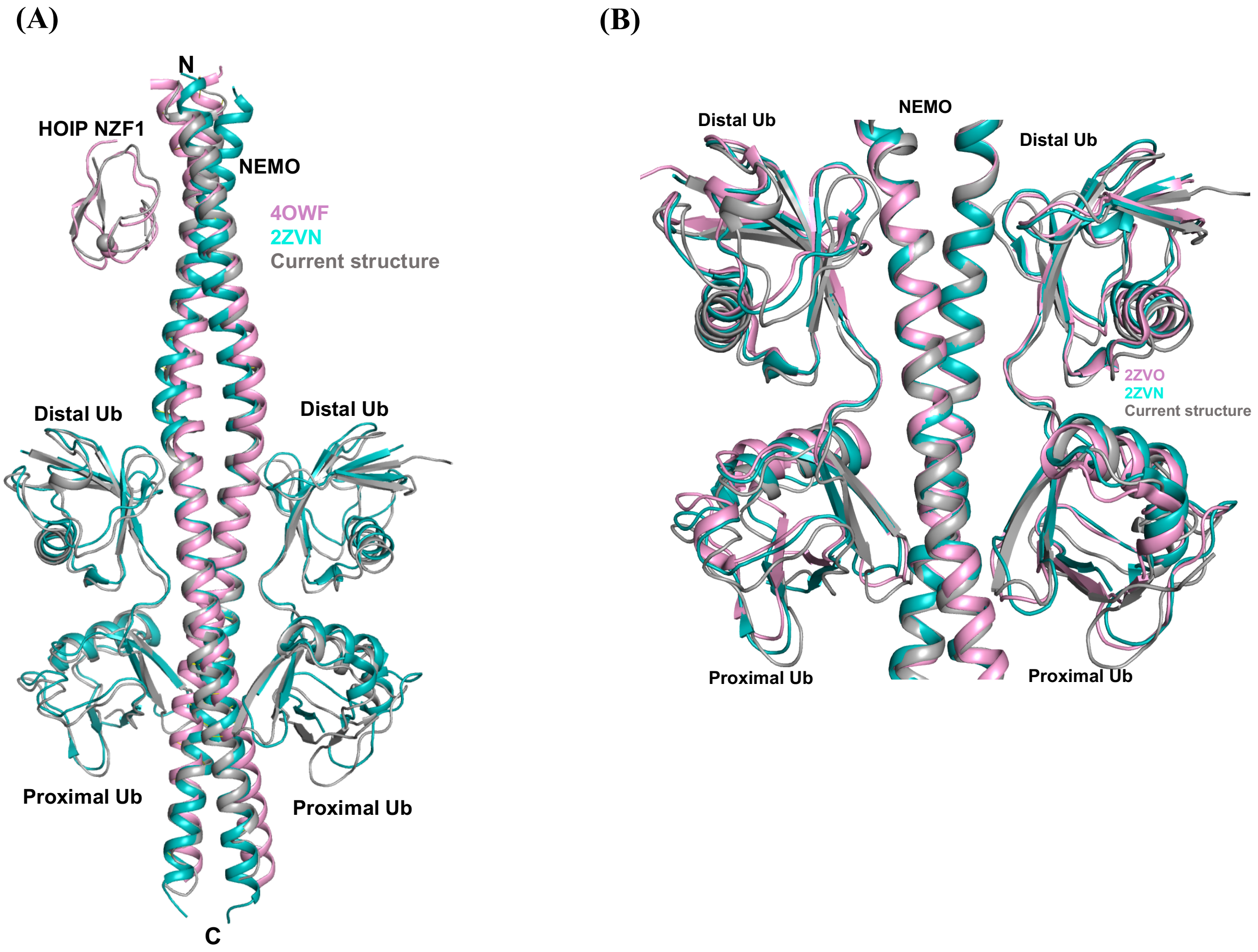

Supplementary Figure S1. NEMO in the heteropentameric structure adopts the same binding mode for HOIP-NZF1 and linear diubiquitins as in the heterotrimeric and heterotetrameric structures. (A) Superimposition of the Cα atoms of NEMO CoZi in the heteropentameric crystal structure (Gray) on NEMO in complex structures containing HOIP NZF1 (PDB ID: 4OWF, pink) and diubiquitions (PDB ID: 2ZVN, cyan). (B) Superimposition of the Cα atoms of UBAN in the heteropentameric structure (Gray) on the UBAN in crystal structures containing linear diubiquitins (2ZVO and 2ZVN).

Supplementary Figure S2

(A)

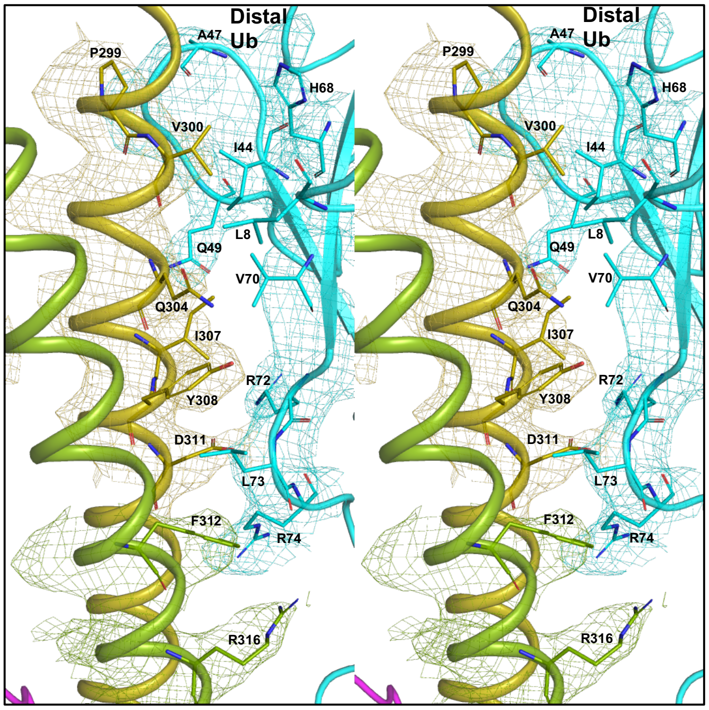

(B)

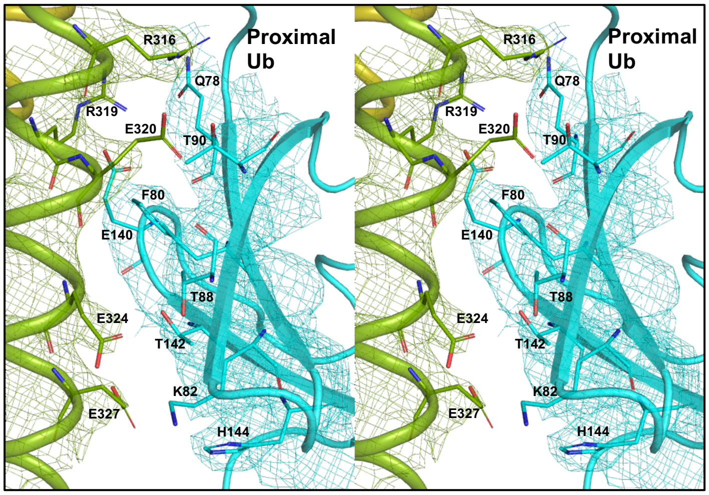

(C)

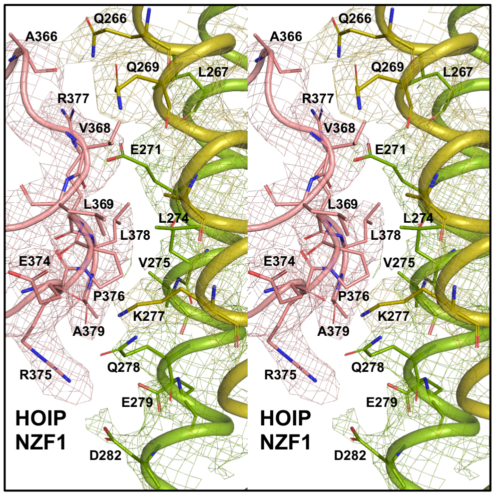

Supplementary Figure S2. Stereo-view of interactions of NEMO with distal (**A**) and proximal (**B**) ubiquitin in a linear diubiquitin chain, and NEMO with HOIP NZF1 (**C**). The final 2Fo - Fc electron density map is contoured at 1.0 σ.

Supplementary Figure S3

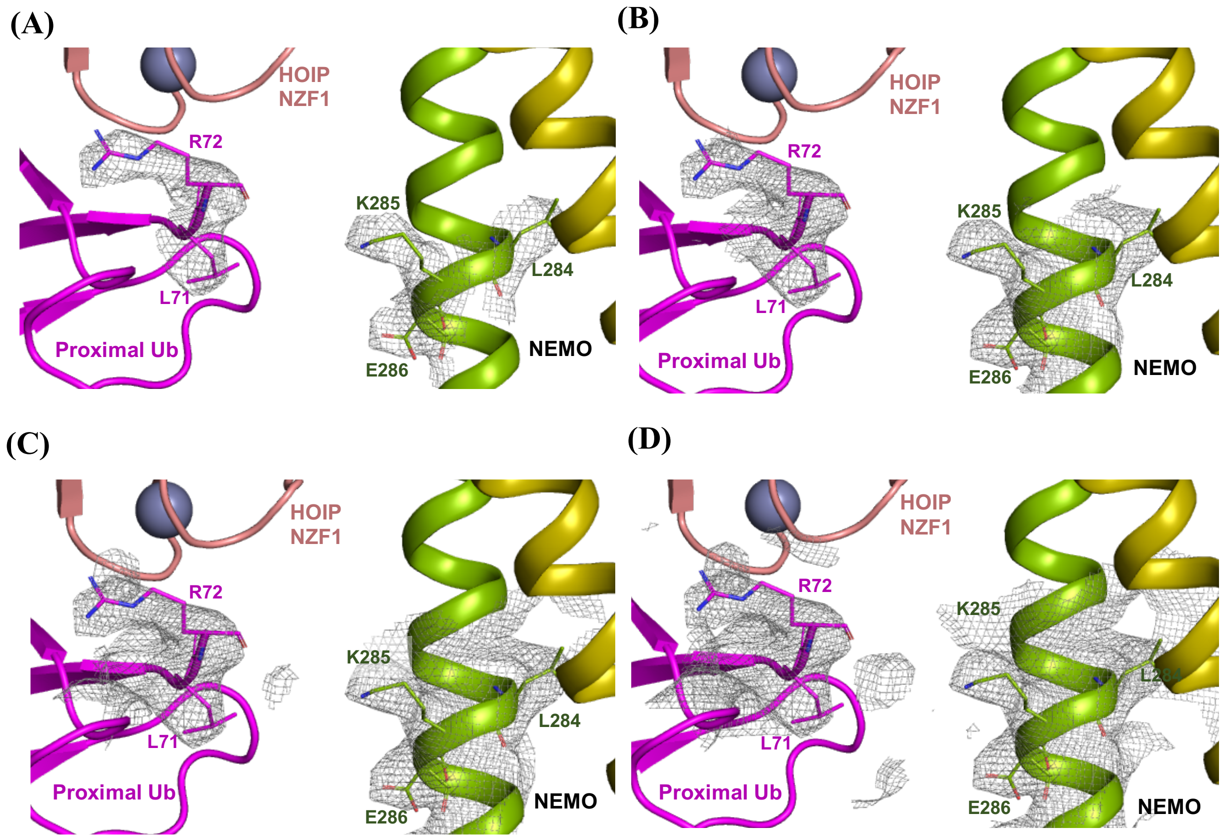

Supplementary Figure S3. The C-terminal tail of proximal ubiquitin, including residues L73, R74, G75, and G76, is not visible in the electron density map. (A) to (D) represent the 2Fo - Fc electron density map contoured at 1.0 σ , within 1.6 Å, 3.0 Å, 4.0 Å, and 5 Å of the L71-R72 of proximal ubiquitin and L284-E286 of NEMO, respectively.
